# Supplementary material for: Evidence of a Set of Core-Function Genes in 16 Bacillus Podoviral Genomes with Considerable Genomic Diversity
Source: Viruses. 2023 Jan 18;15(2):276. doi: 10.3390/v15020276 (PMC9965433; doi:10.3390/v15020276)
Supplement: Supplementary file 1 [file viruses-15-00276-s001.zip › Supplementary Figure legends.pdf]

Supplementary Figure S1. Phamerator map of podoviral genomes. Phage genomes are grouped according to dotplot and ANI derived groupings. Proteins are represented by rectangles along the genome rulers, with labels identifying protein phams and the number of representatives in each pham. Proteins with predicted conserved domains are indicated by yellow bars inside the protein rectangle. Shading between genomes indicates the level of nucleotide sequence similarity, with purple shading having the highest similarity and E-value by blastn.

Supplementary Figure S2. pRNA sequence range and tertiary fold predictions. Locations of predicted pRNA sequences from the literature and this study are listed, along with predicted tertiary folds for select sequences (Domain 1 only). Conserved nucleotides (green) as well as nucleotides that may participate in intermolecular interactions (red) are colored according to [27].
