# Supplementary material for: Evidence of a Set of Core-Function Genes in 16 Bacillus Podoviral Genomes with Considerable Genomic Diversity
Source: Viruses. 2023 Jan 18;15(2):276. doi: 10.3390/v15020276 (PMC9965433; doi:10.3390/v15020276)
Supplement: Supplementary file 1 [file viruses-15-00276-s001.zip › Supplementary Figure S1 Phamerator maps of phage genomes.pdf]

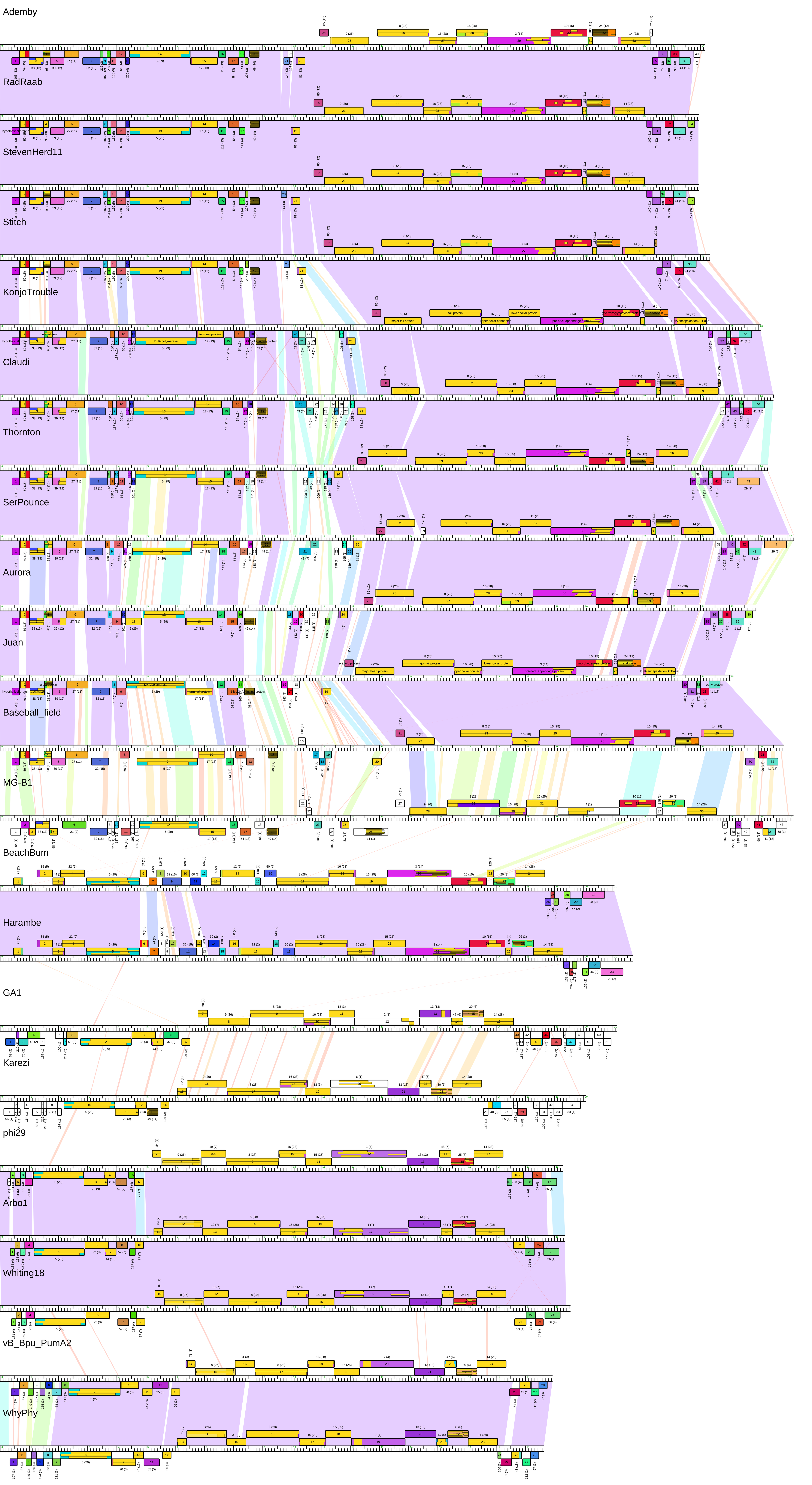
Ademby
RadRaab
StevenHerd11
Stitch
KonjoTrouble
Claudi
Thornton
SerPounce
Aurora
Juan
Baseball_field
MG-B1
BeachBum
Harambe
GA1
Karezi
phi29
Arbo1
Whiting18
vB_Bpu_PumA2
WhyPhy
