# Supplementary material for: Evidence of a Set of Core-Function Genes in 16 Bacillus Podoviral Genomes with Considerable Genomic Diversity
Source: Viruses. 2023 Jan 18;15(2):276. doi: 10.3390/v15020276 (PMC9965433; doi:10.3390/v15020276)
Supplement: Supplementary file 1 [file viruses-15-00276-s001.zip › Supplementary Figure S2 prna prediction.pdf]

Phage	Ref.	pRNA range	Length
GA-1	[51]	1879-1719	160
phi29	[50]	321-147	174
Ademby	this study	365-203	163
RadRaab	[52]	375-213	163
StevenHerd11	this study	374-212	163
Stitch	[52]	375-213	163
KonjoTrouble	[52]	375-213	163
Claudi	[52]	376-214	163
Thornton	this study	376-214	163
SerPounce	[52]	378-216	163
Aurora	[52]	378-216	163
Juan	[52]	375-213	163
BeachBum	[52]	288-122	166
Harambe	[52]	288-122	166
Karezi	this study	1075-911	164
WhyPhy	this study	212-23	189
Whiting	this study	352-179	174
Arbo1	this study	314-174	174

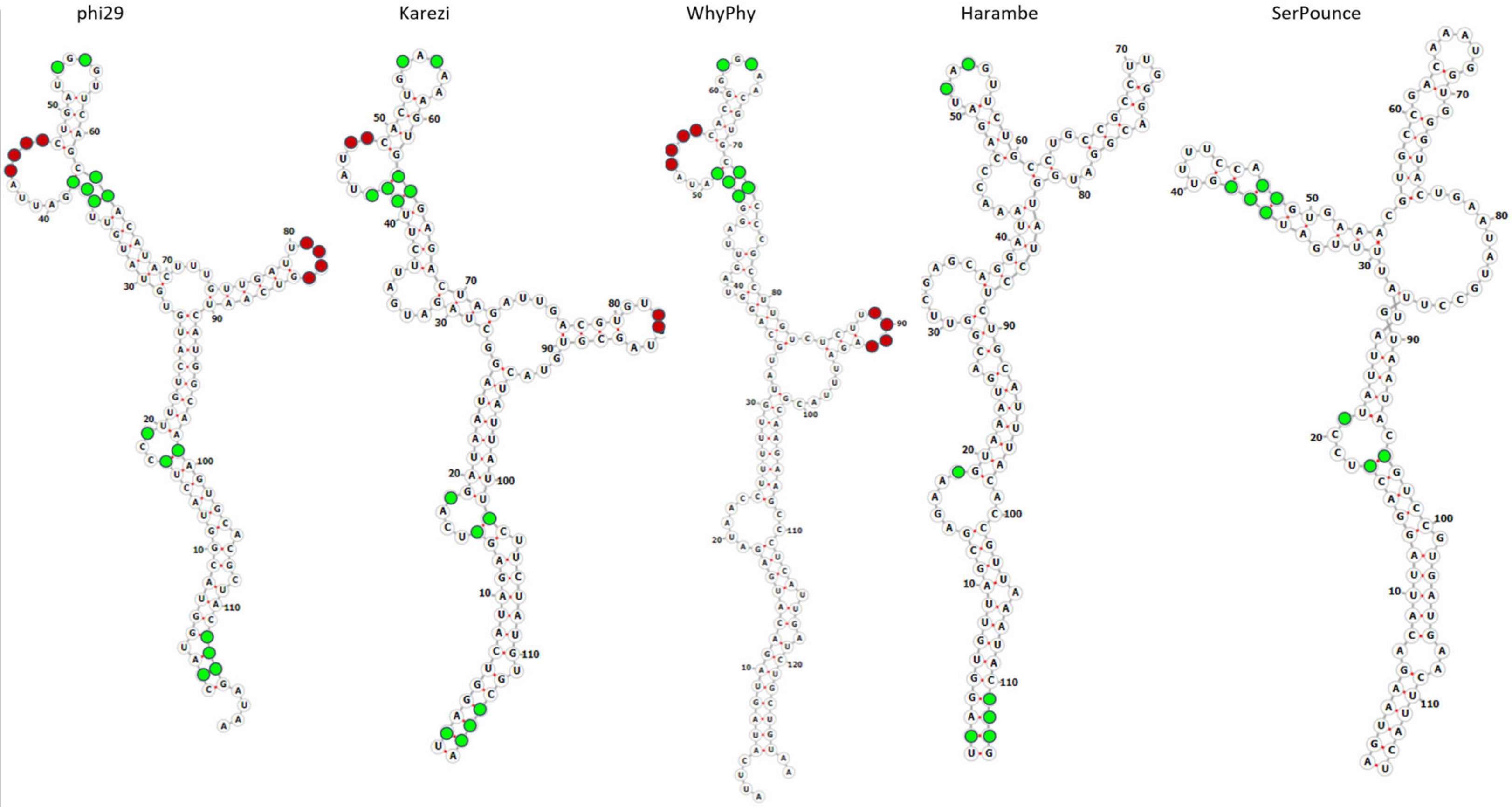

Supplementary Figure S2
