# Supplementary material for: Evidence of a Set of Core-Function Genes in 16 Bacillus Podoviral Genomes with Considerable Genomic Diversity
Source: Viruses. 2023 Jan 18;15(2):276. doi: 10.3390/v15020276 (PMC9965433; doi:10.3390/v15020276)
Supplement: Supplementary file 1 [file viruses-15-00276-s001.zip › Supplementary Table S1.pdf]

Supplemental Table S1: Average Nucleotide Identity

	Ademby	RadRaab	StevenHerd11	Stitch	Thornton	Claudi	KonjoTrouble	SerPounce	Aurora	Baseball_field	DK1	DLc1	MG-B1	BeachBum	Harambe	VMY22	Whiting18	Arbo	phi29	B103	Karezi	GA1	WhyPhy	PumA2	Cp1
**Ademby**		99	99	99	91	90	90	89	87	90	75	74	73	61	60	58	56	56	55	57	56	57	57	56	52
**RadRaab**	99		100	99	90	90	90	89	87	90	76	72	74	60	60	58	57	57	56	57	56	57	57	57	51
**StevenHerd11**	99	100		99	90	90	90	89	87	90	76	72	74	60	60	58	56	57	56	57	56	57	57	57	51
**Stitch**	99	99	99		91	91	90	89	87	91	75	74	74	61	60	58	56	56	56	57	56	57	57	56	51
**Thornton**	91	90	90	91		96	96	94	88	95	76	73	74	60	60	57	56	56	56	57	55	57	56	56	52
**Claudi**	90	90	90	91	96		96	94	88	96	76	72	74	60	59	57	0	55	55	57	55	57	56	56	51
**KonjoTrouble**	90	90	90	90	96	96		93	88	95	76	73	74	59	59	57	57	57	55	56	56	57	57	56	51
**SerPounce**	89	89	89	89	94	94	93		88	96	77	72	75	60	60	57	56	57	56	57	56	57	57	56	52
**Aurora**	87	87	87	87	88	88	88	88		88	76	72	73	60	60	58	57	57	56	57	56	56	56	55	52
**Baseball_field**	90	90	90	91	95	96	95	96	88		76	72	74	60	60	57	56	56	55	57	56	56	57	56	52
**DK1**	75	76	76	75	76	76	76	77	76	76		73	73	60	59	58	55	55	55	56	56	56	57	58	51
**DLc1**	74	72	72	74	73	72	73	72	72	72	73		72	60	60	58	56	56	56	57	55	56	57	56	52
**MG-B1**	73	74	74	74	74	74	74	75	73	74	73	72		59	59	58	57	57	56	58	56	56	56	57	52
**BeachBum**	61	60	60	61	60	60	59	60	60	60	60	60	59		96	60	56	55	56	58	55	56	57	57	52
**Harambe**	60	60	60	60	60	59	59	60	60	60	59	60	59	96		60	56	56	56	58	56	57	56	57	52
**VMY22**	58	58	58	58	57	57	57	57	58	57	58	58	58	60	60		58	58	57	58	56	56	57	56	52
**Whiting18**	56	57	56	56	56	55	57	56	57	56	55	56	57	56	56	58		99	90	68	57	57	58	56	51
**Arbo**	56	57	57	56	56	55	57	57	57	56	55	56	57	55	56	58	99		90	68	57	57	58	57	51
**phi29**	55	56	56	56	56	55	55	56	56	55	55	56	56	56	56	57	90	90		68	57	57	58	58	51
**B103**	57	57	57	57	57	57	56	57	57	57	56	57	58	58	58	58	68	68	68		57	58	58	58	51
**Karezi**	56	56	56	56	55	55	56	56	56	56	56	55	56	55	56	56	57	57	57	57		64	57	57	50
**GA1**	57	57	57	57	57	57	57	57	56	56	56	56	56	56	57	56	57	57	57	58	64		58	58	52
**WhyPhy**	57	57	57	57	56	56	57	57	56	57	57	57	56	57	56	57	58	58	58	58	57	58		86	51
**PumA2**	56	57	57	56	56	56	56	56	55	56	58	56	57	57	57	56	56	57	58	58	57	58	86		51
**Cp1**	52	51	51	51	52	51	51	52	52	52	51	52	52	52	52	52	51	51	51	51	50	52	51	51	
